# Supplementary figures and images for: LINE-1 transposition into murine Thyroglobulin results in congenital thyroid dysplasia
Source: PLoS One. 2025 Jul 8;20(7):e0325493. doi: 10.1371/journal.pone.0325493 (PMC12237065; doi:10.1371/journal.pone.0325493)

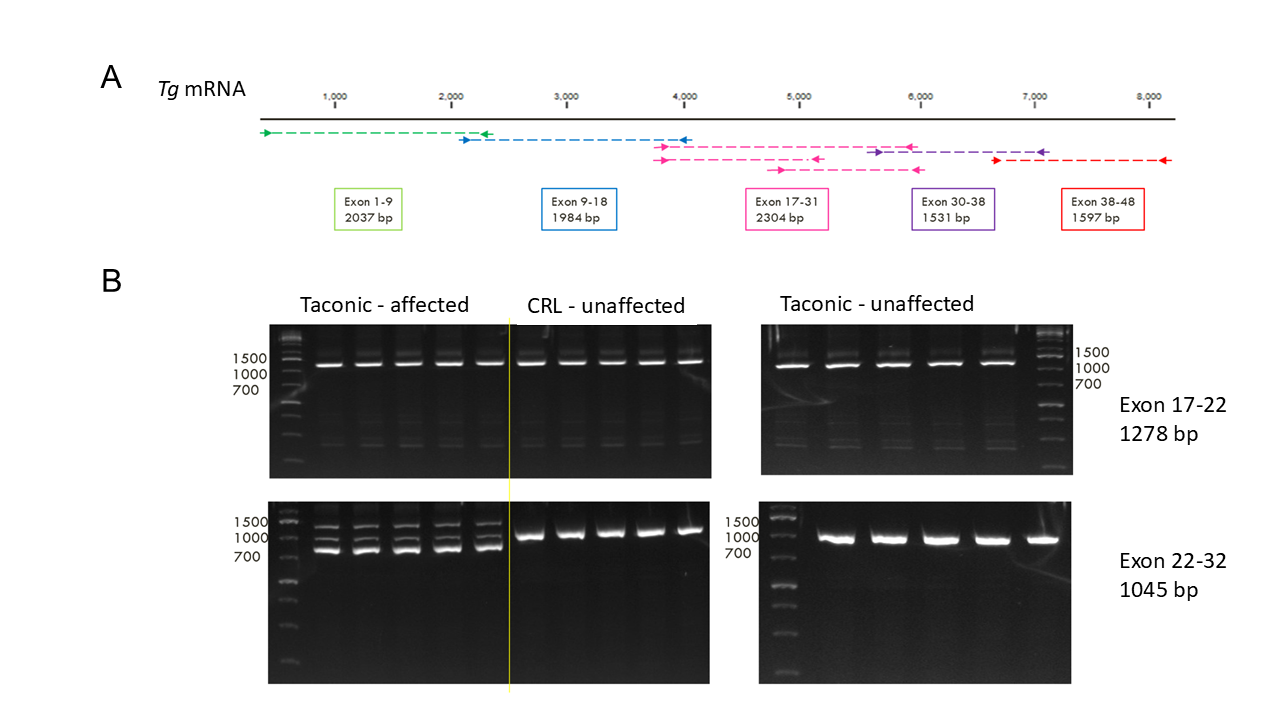

Supplement: S1 Fig — (A) Schematic representation of the full-length Tg mRNA (solid line) displaying the overlapping amplicons (dashed arrows). (B) Gel electrophoresis images showing amplification results of the amplicon covering exon 17–22 (upper panels) and amplicon covering exon 22–32 (lower panels). (TIF) [file pone.0325493.s001.tif]

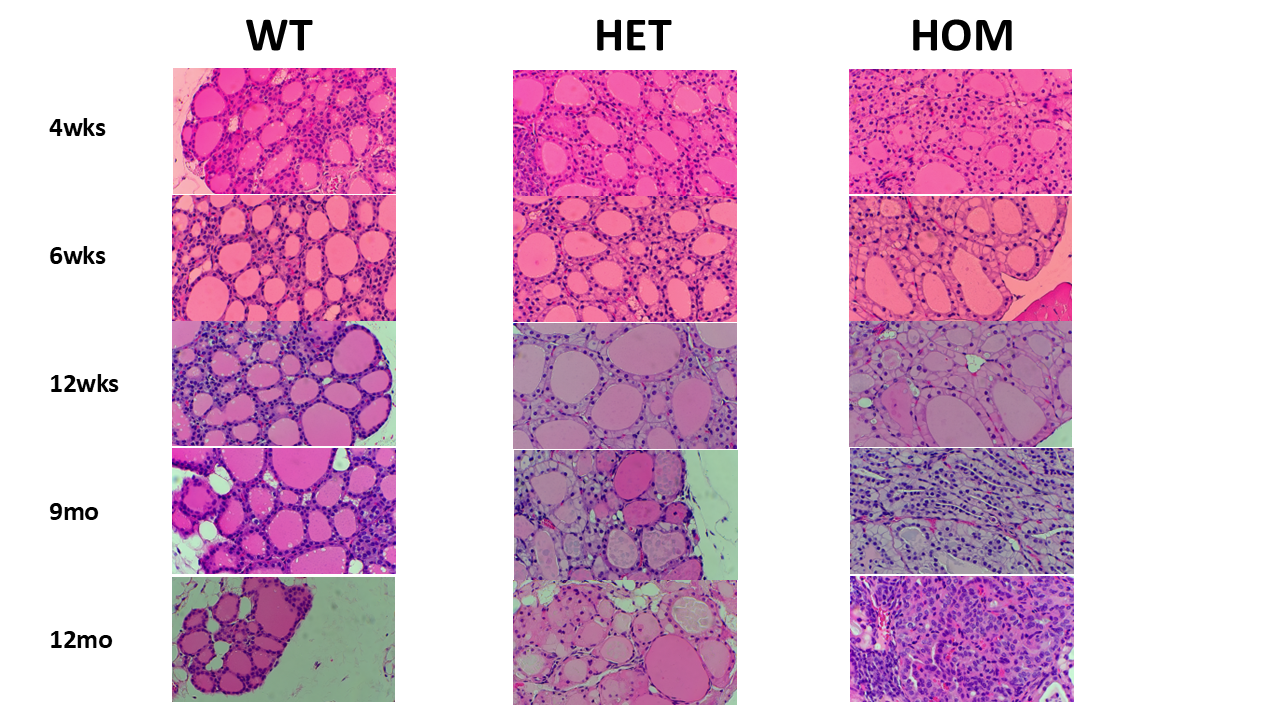

Supplement: S2 Fig — Shown are representative microscopic images of H&E-stained thyroid follicular epithelial cells from B6NTac animals WT, HET, or HOM for the LINE-1 in Tg at 4 wks, 6 wks, 12 wks, 9 months, and 12 months of age. (TIF) [file pone.0325493.s002.tif]

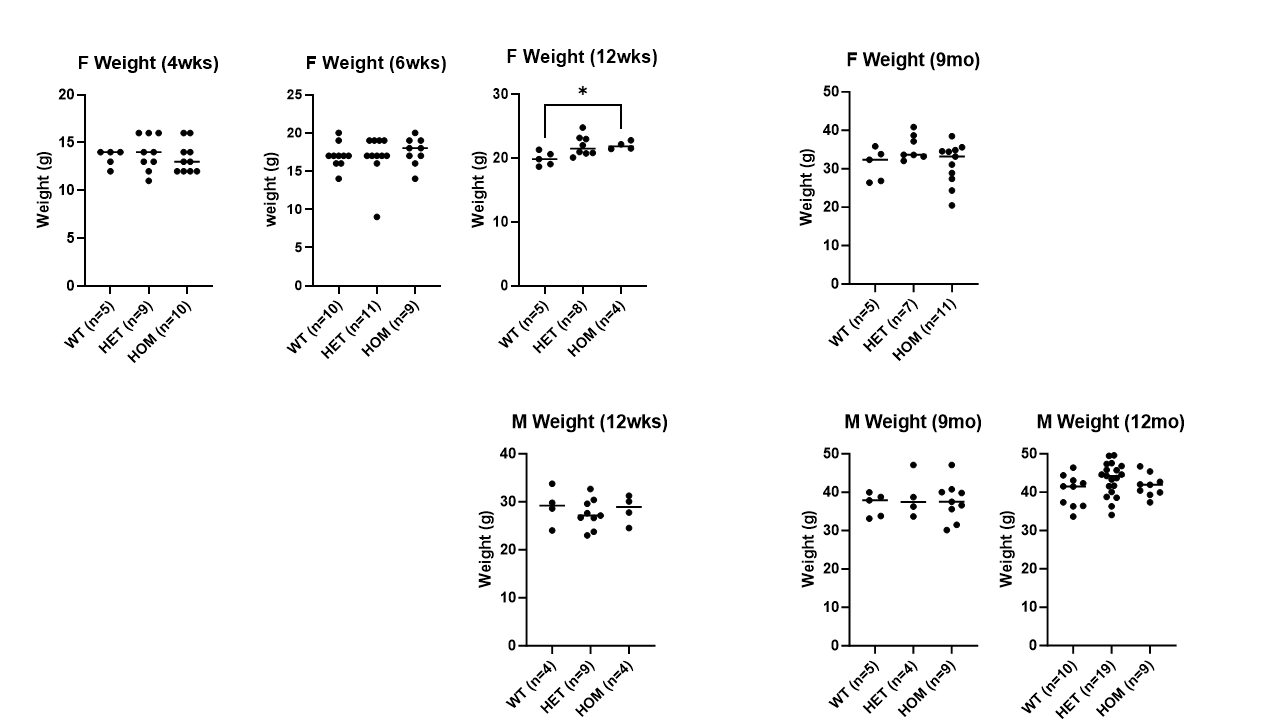

Supplement: S3 Fig — BW measurements of B6NTac animals WT, HET and HOM for the LINE-1 in Tg at 4 wks, 6 wks, 12 wks, 9 mo and 1 yr of age, split by sex. The data were analyzed by One-way ANOVA with Bonferroni correction. Significance is indicated by * (adj. p < 0.05). (TIF) [file pone.0325493.s003.tif]

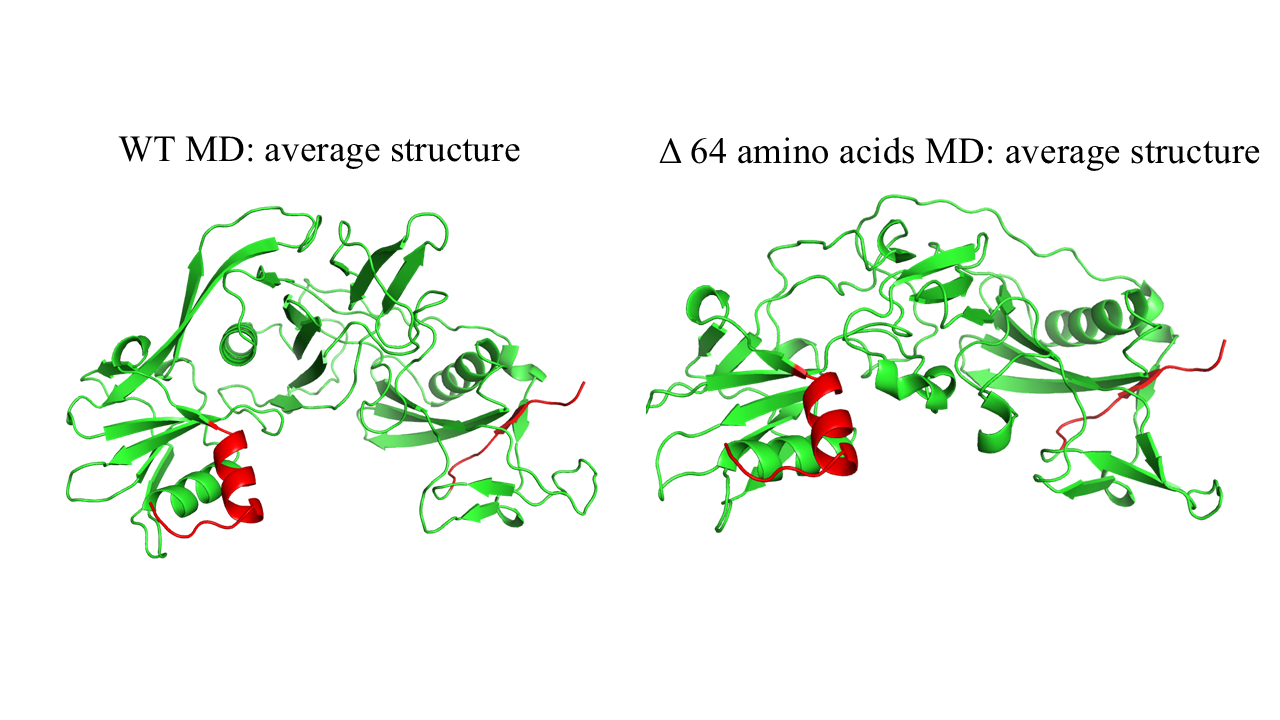

Supplement: S4 Fig — The 15 N-terminal and 9 C-terminal segments with positional restraints placed during simulation were colored as red. (TIF) [file pone.0325493.s004.tif]
