# Supplementary material for: LINE-1 transposition into murine Thyroglobulin results in congenital thyroid dysplasia
Source: PLoS One. 2025 Jul 8;20(7):e0325493. doi: 10.1371/journal.pone.0325493 (PMC12237065; doi:10.1371/journal.pone.0325493)
Supplement: Attachment — Submitted filename: Response to Reviewers.docx [file pone.0325493.s009.docx]

**Editor’s Comments with Author’s Responses and Rebuttals:**

When submitting your revision, we need you to address these additional requirements.

1.Please ensure that your manuscript meets PLOS ONE's style requirements, including those for file naming. The PLOS ONE style templates can be found at

<https://journals.plos.org/plosone/s/file?id=wjVg/PLOSOne_formatting_sample_main_body.pdf> and

[https://journals.plos.org/plosone/s/file?id=ba62/PLOSOne_formatting_sample_title_authors_affiliations.pdf](https://track.editorialmanager.com/CL0/https:%2F%2Fjournals.plos.org%2Fplosone%2Fs%2Ffile%3Fid=ba62%2FPLOSOne_formatting_sample_title_authors_affiliations.pdf/1/010f01951b95b28c-180f38aa-9945-4333-b981-a69f3cc46496-000000/zoiw49IqhvYLakWQbGP9rEac3DpKkYQK6Q3nVPDr4X0=199)

To the best of our ability we have formatted the manuscript, figures and tables in the PLOS ONE style.

2. To comply with PLOS ONE submissions requirements, in your Methods section, please provide additional information regarding the experiments involving animals and ensure you have included details on methods of anesthesia and/or analgesia, and efforts to alleviate suffering.

We added the following clarification about anesthesia in the methods section:

*“Euthanasia was performed by CO2 asphyxiation, followed by cervical dislocation as a secondary method.”*

With respect to alleviating suffering, the methods section includes the following statements confirm that both Taconic and Merck comply with IACUC standards and NIH Guidelines.

*“All mice were kept in an AAALAC-accredited facility at Taconic Biosciences. All procedures have been approved by Taconic Biosciences’ Institutional Animal Care and Use Committee (IACUC).”*

*“All animal procedures conducted at Merck & Co., Inc., Rahway, NJ, USA were approved by the Merck & Co., Inc., Rahway, NJ, USA Institutional Animal Care and Use Committee and conducted in an Association for Assessment and Accreditation of Laboratory Animal Care International–accredited facility in compliance with the National Institutes of Health (NIH) Guide for the Care and Use of Laboratory Animals and the Animal Welfare Act.”*

3. We note that your Data Availability Statement is currently as follows: “All relevant data are within the manuscript and in Supporting Information files.”

Please confirm at this time whether or not your submission contains all raw data required to replicate the results of your study. Authors must share the “minimal data set” for their submission. PLOS defines the minimal data set to consist of the data required to replicate all study findings reported in the article, as well as related metadata and methods (<https://journals.plos.org/plosone/s/data-availability#loc-minimal-data-set-definition>).

For example, authors should submit the following data:

- The values behind the means, standard deviations and other measures reported;

- The values used to build graphs;

- The points extracted from images for analysis.

Authors do not need to submit their entire data set if only a portion of the data was used in the reported study.

If your submission does not contain these data, please either upload them as Supporting Information files or deposit them to a stable, public repository and provide us with the relevant URLs, DOIs, or accession numbers. For a list of recommended repositories, please see <https://journals.plos.org/plosone/s/recommended-repositories>.

Raw data used to generate all the plots and figures have been uploaded as a Supporting Information file (S2 File).

We are in the process of submitting raw sequencing reads from the RNAseq analysis to GEO. The accession number is still pending. We entered ‘GEOxxxx’ in the text as a placeholder.

If there are ethical or legal restrictions on sharing a de-identified data set, please explain them in detail (e.g., data contain potentially sensitive information, data are owned by a third-party organization, etc.) and who has imposed them (e.g., an ethics committee). Please also provide contact information for a data access committee, ethics committee, or other institutional body to which data requests may be sent.

There are no ethical or legal restrictions on sharing any of the data in this manuscript. Data access requests can be directed to the corresponding author at email: [bart.smits@taconic.com](mailto:bart.smits@taconic.com)

4. Please include captions for your Supporting Information files at the end of your manuscript, and update any in-text citations to match accordingly. Please see our Supporting Information guidelines for more information: <http://journals.plos.org/plosone/s/supporting-information>.

We have inserted the Supporting Information captions at the end of the manuscript and confirmed that in-text citations match. The Supporting Figures have been submitted as separate files.

**Additional Editor Comments:**

1) How line-1 insertion affect splicing mechanism in the context of cis-acting regulation or spliceosome have not been explored

We thank the Editor for this thoughtful comment. We refer to our response to the Academic Reviewer’s question regarding the mechanism of exon-skipping.

2) Effect of UPR on metabolic dysregulation, apoptosis and cell stress are explored

Correct, effects of the UPR on metabolic dysregulation, apoptosis and cell stress are addressed in the discussion on page 19 of the manuscript.

3) Authors also did not show how mutation affect other gene or signalling pathway

Thank you for raising this point. We conducted transcriptome pathway analysis to address potential effects on other pathways and discussed the biological relevance of those results in the context of the LINE-1 insertional event. The activation of an unfolded protein chaperon response network found with this analysis was confirmed by TEM used to characterize the misfolded Tg proteins in the ER of thyroid follicular epithelial cells of affected animals. See section Molecular modeling of variant TG protein indicates that the omission of 64 amino acids impacts stability on page 11, in the Discussion section on page 18 and in Table 1. The analysis did not identify other compensatory pathways with significant p-values that would explain any of the hormonal or histopathological findings.

4) The mutation is stably inherited across generation?

Yes, this is a germ line mutation that is inherited in an autosomal dominant manner, as described on page 9 and in Fig. 3. All 3 HOM and all 4 HET pups as well as both HET parents showed thyroid dysplasia and the WT pups did not, indicating that the phenotype is strongly associated with the LINE-1 in Tg and inherited in a dominant fashion. We actually found presence of the LINE-1 in frozen stock from the B6Ntac colony established in 2014, suggesting that the actual retrotransposition event occurred before that time. We added this fact in the results section on page 9.

5) Authors also didn’t mention any therapeutic intervention.

Thank you for bringing up therapeutic intervention. We feel that clinical relevance of this mutation is out of scope of our current work, since to our knowledge there is no known LINE-1 mutation in human Thyroglobulin. On page 16 in the discussion we do comment on the potential for hidden histological phenotypes for heterozygous carriers of Thyroglobulin mutations. Please also refer to the response to point 2 raised by the Academic Reviewer.

**Reviewers Comments and Rebuttal:**

1. Could you elaborate on how the LINE-1 insertion specifically disrupts splicing at the molecular level? Are there cryptic splice sites involved?

We thank the reviewer for this thoughtful question. While investigation into the exact mechanism of how splicing was affected by the LINE-1 insertional mutation is beyond the scope of our current work and should be investigated in more detail in a future study, we hypothesized a mechanism that the LINE-1 insertion into the intron affects pre-mRNA branching and effective splicing at exon 26. Additionally, we re-analyzed the RNAseq data and determined that exon-skipping without significant exonization of the intron due to a cryptic splice site is the most plausible hypothesis.  Future experiments should focus on quantification of the contribution of various exon-skipping mechanism to the *Tg* transcript load.  Please see the revised content in the discussion section that serves to address this comment on pages 17-18.

2. Are there any known human analogs of this mutation? How does this model reflect human thyroid dysplasia mechanisms?

We thank the reviewer for bringing up this point to discuss. We added the following section to the discussion on page 19:

“There are no published human analogs of this mutation in the thyroglobulin gene despite many well documented instances of diseases resulting from transposable element insertions ([Hancks and Kazazian 2016](https://pmc.ncbi.nlm.nih.gov/articles/PMC4859970/)), numerous mutations resulting in thyroid disease and cancer ([Kostapoulou et al, 2021](https://pubmed.ncbi.nlm.nih.gov/33400193/), [Romei and Elisei 2021](https://pubmed.ncbi.nlm.nih.gov/33572167/)) and numerous Thyroglobulin mutations causing thyroid disfunction (Citterio et al., 2021). One reason for this may be that humans have evolved more effective “defense” mechanisms to suppress TE mobilization and/or the relatively rapid generation time in mice allowing more opportunities for LINE-1 activation and random insertional events ([Goodier 2016](https://pmc.ncbi.nlm.nih.gov/articles/PMC7778262/#GAD344473HERC59); [Oomen 2025](https://r.search.yahoo.com/_ylt=AwrFdi..9t5ntQIA29pXNyoA;_ylu=Y29sbwNiZjEEcG9zAzEEdnRpZAMEc2VjA3Ny/RV=2/RE=1743875007/RO=10/RU=https%3a%2f%2fwww.sciencedirect.com%2fscience%2farticle%2fpii%2fS0092867424014260/RK=2/RS=AcLT9geS1T3pB5UZvgPKZL86V_I-)). Another reason might be that the human genome has only ~100 full-length, active LINE-1, while the mouse genome has ~3,000. Therefore, the chance that a LINE-1 mobilizes and lands in any gene is much higher in the mouse genome than in the human genome. Thus, the subtle physiological effect of the intronic LINE-1 identified in this study can only be recapitulated in human if a similar mutation arises in the human intron 25 causing exon-skipping at incomplete penetrance, which to our knowledge has not been identified yet.“

3. What factors might account for the variability in phenotype severity between heterozygous and homozygous mice?

This is discussed under results section Thyroid dysplasia is inherited in an autosomal dominant fashion and is strongly associated with a LINE-1 on pg 8-9. Variability of phenotype severity is considered to be due to different relative amounts of full lenth functional proteins among WT, HETs and HOMS: “The proportion of exon 26-included transcripts in WT was 1.049, in HET was 0.662 and in HOM was 0.245 (Fig. 3b), indicating that HETs and HOMs respectively produce 63% and 23% of the level of exon 26-included transcripts as compared to WT.” The differences of physiological effects as a result of these differences in full length Tg transcript could be considered a gene dosage effect.

We also revised the Discussion section on page 15 as follows: “In the mice evaluated, thyroid follicular cell adenoma was only observed in mice homozygous for LINE-1 retrotransposon insertion but not in the heterozygous mice even up to 18 months of age. We attributed this difference in incidence of adenoma to increased exon 26 skipping in homozygotes leading to decreased full length Tg transcript level. We propose that the gene dosage effect of a greater decrease in full length Tg transcript level would lead to a greater decrease in THs, a concomitant increased TSH release from the pituitary and therefore increased stimulation of thyroid follicular cells to proliferate in homozygotes compared to heterozygotes. This is supported by the observation that heterozygotes have a smaller decrease in serum THs and do not have the increased serum TSH level observed in homozygotes.”

4. Based on your findings, are there specific therapeutic strategies you foresee for conditions involving LINE-1-induced dysregulation?

Thank you for bringing up therapeutic intervention. We feel that clinical relevance of this mutation is out of scope of our current work, since to our knowledge there is no known LINE-1 mutation in human Thyroglobulin as discussed in point 2 above. On page 16 in the discussion we do comment on the potential for hidden histological phenotypes for heterozygous carriers of Thyroglobulin mutations.

5. Have you considered exploring compensatory mechanisms in heterozygous carriers that prevent progression to adenoma?

We hypothesize that the relatively mild effects in heterozygotes are mostly the result of sufficient full length Thyroglobulin transcript and protein being produced. While the mutant form of TG does induce a milder form of thyroid dysplasia, there could be enough full length TG to sustain T3 and T4 production, thereby preventing the negative feedback mechanism involving TSH production from the pituitary gland from being affected. We currently do not have any evidence for the involvement of another compensatory mechanism. The gene dosage effect of the LINE-1 on heterozygotes and homozygotes is discussed on page 15 and 18.

The statistical analysis in the paper is moderately clear and appropriate for validating key findings, but there are areas of improvement. The study makes use of unpaired t-tests in comparing serum hormone levels, T3, T4, and TSH across genotypes, and molecular dynamics metrics like RMSF for assessing protein stability, which are suitable for their respective analyses. Results are described with proper p values (e.g., (p = 0.02, p < 0.01), providing a precise foundation for assertions.

The work is not transparent about other essentials, including how power analyses explain sample sizes for each experiment or confidence intervals plus effect sizes, which would help further contextualize their findings. While sample sizes sometimes vary between experimental conditions, even more importantly and unfortunately, all experiments lacked multiplicity adjustments. The biological import of statistically significantly different values in hormones, such as hormone concentration levels, should also be appreciated better. Overlayers, such as raw data or error bars, onto graphical representations are useful for highlighting data distribution better. These changes will strengthen analysis through more specified statistical methodologies about the study adjusted for multiple comparison tests and providing clearer data illustrations.

We thank the reviewer for bringing up improvements to our statistical methods. We replaced the unpaired t-test analysis with one-way ANOVA analysis with Bonferroni corrections for pair-wise comparisons of the means of each genotype within an age cohort. The following text was modified in the Methods section on page 23: “Levels were imported into GraphPad (Prism) software and groups were compared using one-way ANOVA, followed by the Bonferroni correction for multiple comparisons. Significance was determined at an adjusted p-value of 0.05 or less.”

Figures 3, 5 and S3 were modified to include individual animal data overlays and statistical significance indicated at the adjusted p<0.05. All raw data and statistical analysis are included in S2 File.

To estimate the required sample size for the thyroid hormone measurements, we conservatively estimated the effect sizes, based on another mouse model of hypothyroidism due to Thyroglobulin mutations ([Zhang](https://pmc.ncbi.nlm.nih.gov/articles/PMC9213252/) et al., 2022). For T3, with an effect size of 0.67, standard deviation of 16, difference between the means of 35 and a sample size of minimally 7 per group, the study had 88% (or higher for greater sample sizes) power to reject the null hypothesis at a Bonferroni adjusted p-value of 0.05. For T4, with an effect size of 0.67, standard deviation of 0.8, difference between the means of 1.9, and a sample size of minimally 7 per group, the study had 81% (or higher for larger sample sizes) power to reject the null hypothesis at a Bonferroni adjusted p-value of 0.05. This was added to the statistics section in the Methods on page 23.

I have a few suggestions for the strengthening of the rationale of research. Although the study manages to point out exon skipping as the critical event giving rise to the observed thyroid dysplasia, it is unclear precisely what the molecular mechanism could be by which the LINE-1 insertion impacts splicing. Further experimentation to discern whether cryptic splice sites or other mechanisms are involved would deepen mechanistic understanding. In addition, more comparison to other Tg-related mutations in mice or humans would give context and emphasize this model's uniqueness and limitations. Lastly, the broader implications of the findings could be expanded on, especially how this model might inform understanding of human thyroid disease and other conditions that may be caused by LINE-1 or similar retrotransposons.

We thank the reviewer for these thoughtful suggestions to strengthen the rationale. We feel we have responded in great detail to each point raised in the discussions above (specifically in points 1, 2, 4 and 5) and incorporated additional discussion to the manuscript.

The study offers valuable insights into the structural effects of exon 26 deletion in the TG protein through molecular dynamics (MD) simulations. Nevertheless, there are several areas that could be enhanced in the analysis. First, the focus is on the arm fragment rather than the full-length protein, thereby limiting the context of the simulation. The entire TG protein should be simulated in future studies to capture the more comprehensive structural and functional implications of the mutation. Validation of the computational results through experimental techniques such as cryo-EM or NMR would add more credibility to the findings. The study only reports RMSF for assessing flexibility but would benefit by including RMSD analysis as well. RMSD would offer more accurate results of general structural stability and corresponding conformational shifts, making the conclusions drawn in terms of the mutation's impact on TG stability stronger.

We appreciate the reviewer’s question about modeling the entire Tg protein, but full length Tg protein modeling and validation by cryo-EM or NMR was not feasible at this time. The following RMSD analysis was added in the manuscript on page 13 in the results section and included in Fig. 6, panel E: “Plotting the root-mean squared deviation (RMSD) of MD snapshots against the corresponding first frame also revealed a larger conformational shift for the mutant as time progressed (Fig. 6E), further indicating the compromised stability due to exon 26 skipping*.”*

It would be great if this could be conducted to explore how the observed structural changes translate to functional consequences for TG, including their potential impact on thyroid hormone synthesis. Further interaction of mutant TG with cellular factors like chaperones is a way to get a more comprehensive understanding of the effects that the mutation could have.

We thank the reviewer for this thoughtful comment. Coscia et al ([Nature 2020](https://pmc.ncbi.nlm.nih.gov/articles/PMC7170718/)) published the structure of the human TG protein which indicates that the arm plays a critical structural role with the central ChEL dimer interacting with different sub-regions within the arm. Our computational analysis of the mutant protein structure in combination with the IHC and EM analysis showing Tg build-up in the ER indicates that the absence of exon 26 from the transcript causes a general destabilization of the protein scaffold that results in misfolding within the ER, resulting in an increased UPR. We acknowledge that cryo-EM or NMR would serve to validate this hypothesis on the molecular level, which could be done in future studies.
