# Supplementary material for: LINE-1 transposition into murine Thyroglobulin results in congenital thyroid dysplasia
Source: PLoS One. 2025 Jul 8;20(7):e0325493. doi: 10.1371/journal.pone.0325493 (PMC12237065; doi:10.1371/journal.pone.0325493)
Supplement: Attachment — Submitted filename: Review by Samudra Pal .docx [file pone.0325493.s008.docx]

**Review of Manuscript PONE-D-24-47670**

**Summary**

This manuscript investigates the impact of a LINE-1 insertion in exon 26 of the thyroglobulin (TG) gene on splicing, protein stability, and thyroid function using mouse models. The authors characterize both heterozygous and homozygous phenotypes through RNA-seq re-analysis, qRT-PCR, molecular dynamics (MD) simulations, hormone assays, and transmission electron microscopy. Overall, the study is well conceived, employs appropriate methods, and the authors have satisfactorily addressed all reviewer and editor comments in their revised submission.

**Major Points**

1. **Splicing Mechanism**
   - **Comment:** Elaborate on how the LINE-1 insertion disrupts splicing.
   - **Response & Revision:** The authors added a discussion of potential branch-site interference and ruled out significant cryptic-site exonization based on re-analysis of RNA-seq reads (Discussion, pp. 17–18).
   - **Status:** Adequately addressed.
2. **Human Analogs**
   - **Comment:** Are there known human TG LINE-1 insertions?
   - **Response & Revision:** New paragraph clarifies no reported human analogs and speculates on selective barriers (Discussion, p. 19).
   - **Status:** Adequately addressed.
3. **Genotype–Phenotype Dosage**
   - **Comment:** Account for variability between heterozygotes and homozygotes.
   - **Response & Revision:** Added quantitative RT-PCR data showing 63% vs. 23% exon 26 inclusion in HET vs. HOM and a gene-dosage explanation (Results, p. 9).
   - **Status:** Adequately addressed.
4. **Molecular Dynamics Analyses**
   - **Comment:** Include RMSD plots; consider full-length TG modeling.
   - **Response & Revision:** RMSD plot added (Fig. 6E; Results, p. 13); full-length modeling deferred to future work.
   - **Status:** Adequately addressed.
5. **Compensatory Mechanisms in Heterozygotes**
   - **Comment:** Explore compensatory pathways.
   - **Response & Revision:** Discussion reiterates a gene-dosage model and notes no additional pathways detected.
   - **Status:** Adequately addressed.
6. **Statistical Methods**
   - **Comment:** Clarify power analyses, multiple comparisons, effect sizes, and confidence intervals.
   - **Response & Revision:** Replaced t-tests with one-way ANOVA plus Bonferroni corrections (Methods, p. 23); added power calculations for hormone assays.
   - **Status:** Adequately addressed.
7. **Editor’s Requests**
   - **Supporting Information captions** moved to end of manuscript; in-text citations updated.
   - **Ethics and anaesthesia details** added (“CO₂ asphyxiation, followed by cervical dislocation…” in Methods).
   - **Data availability**: Raw data in S2 File with a “GEOxxxx” placeholder.
   - **UPR pathway discussion** and TEM confirmation remain integrated.
   - **Status:** Adequately addressed, pending GEO accession finalization.

**Minor Suggestions Before Final Acceptance**

1. **GEO Accession:** Replace “GEOxxxx” placeholder with the actual accession number once available.
2. **Figure Legends:** Explicitly state “one-way ANOVA with Bonferroni correction” in legends reporting adjusted p-values.
3. **Effect‐Size Reporting:** Consider including confidence intervals or η² values for ANOVA results to contextualize biological significance.
4. **Mechanistic Validation:** If feasible, add targeted RT-PCR assays probing for any rare cryptic splice junctions to strengthen the splicing‐interference hypothesis.

**Recommendation:**
Accept pending minor revisions as outlined above.
